# Is the Use of a Low-Cost sEMG Sensor Valid to Measure Muscle Fatigue?

**DOI:** 10.3390/s19143204

**Published:** 2019-07-20

**Authors:** Sergio Fuentes del Toro, Silvia Santos-Cuadros, Ester Olmeda, Carolina Álvarez-Caldas, Vicente Díaz, José Luís San Román

**Affiliations:** 1 Mechanical Engineering Department, Universidad Carlos III de Madrid, Avda. de la Universidad 30, 28911 Leganés, Spain; 2 Institute for Automotive Vehicle Safety (ISVA), Universidad Carlos III de Madrid, Avda. de la Universidad 30, 28911 Leganés, Spain

**Keywords:** low-cost hardware, electromyography, validation

## Abstract

Injuries caused by the overstraining of muscles could be prevented by means of a system which detects muscle fatigue. Most of the equipment used to detect this is usually expensive. The question then arises whether it is possible to use a low-cost surface electromyography (sEMG) system that is able to reliably detect muscle fatigue. With this main goal, the contribution of this work is the design of a low-cost sEMG system that allows assessing when fatigue appears in a muscle. To that aim, low-cost sEMG sensors, an Arduino board and a PC were used and afterwards their validity was checked by means of an experiment with 28 volunteers. This experiment collected information from volunteers, such as their level of physical activity, and invited them to perform an isometric contraction while an sEMG signal of their quadriceps was recorded by the low-cost equipment. After a wavelet filtering of the signal, root mean square (RMS), mean absolute value (MAV) and mean frequency (MNF) were chosen as representative features to evaluate fatigue. Results show how the behaviour of these parameters across time is shown in the literature coincides with past studies (RMS and MAV increase while MNF decreases when fatigue appears). Thus, this work proves the feasibility of a low-cost system to reliably detect muscle fatigue. This system could be implemented in several fields, such as sport, ergonomics, rehabilitation or human-computer interactions.

## 1. Introduction

Muscle fatigue is a very common occurrence in daily life. In many instances, fatigue can lead to injury. Consequently, if a fatigue detection system could guide a user in his or her training or tasks and provide accurate muscle fatigue level readings, unnecessary strain on muscles could be avoided and thus injuries could also be prevented [1]. Human-computer interfaces using bioelectrical signals as inputs can become valuable tools in order to improve the life quality of people, especially for people who have disabilities or injuries. These interfaces provide communication, situation control and feedback between users and their surroundings. Therefore, it could be very useful to develop systems based on EMG signals, which could detect risk and alert users. Common classes of bio-signals used to control assistive devices are [2]: electromyography (EMG) [3], electroencephalography (EEG) [4], electrooculography (EOG) [5], and fusions of these signals [6].

The benefits of muscle fatigue detection can be implemented in several fields, such as sports medicine and performance, human-computer interactions, ergonomics [7], rehabilitation medicine, physiotherapy, neurophysiology, kinesiology and prosthetics. This wide field of uses is the main reason why this work seeks to demonstrate a procedure to assess and detect muscle fatigue. Furthermore, in order to make this procedure more accessible to more people, we will focus on a low-cost system to carry it out.

Muscle fatigue can be defined as the process of decline of the applied force that a muscle is able to exert during a period of sustained activity. From a physiological point of view, fatigue is further defined as the inability to exert any more force or power [8,9]. During a fatiguing contraction, biological changes happen, such as an increase in metabolite concentrations, changes in muscle fibre conduction velocity, and alterations in the number of motor units recruited [10]. A muscle fatigues due to the accumulation of lactic acid in the muscle tissue and the depletion of glycogen (stored glucose), resulting in a reduction of the muscle’s contractile properties [1]. Directly tracking the physiological processes in muscle contraction is difficult and impractical. Nevertheless, monitoring the surface electromyogram can help us detect electrical manifestations of these physiological events. Myoelectric parameters such as conduction velocity [11], mean frequency, median frequency [9,12] and instantaneous mean frequency [13,14,15] also accurately reflect levels of muscle fatigue.

Current research [1] tends to distinguish localized muscle fatigue in two levels: Non-fatigue and Fatigue. Non-fatigue relates to the muscle state during the contraction that occurs before fatigue begins, while Fatigue alludes to the start of declining muscular capacity during a muscle contraction. Other authors [16] mentioned a third level: Transition-to-fatigue. In the Non-fatigue level, a muscle can apply its maximum force. When the muscle starts to fatigue, there is new recruitment of muscle fibres. This is the onset of Transition-to-fatigue, and this state is a progressive process until the Fatigue level begins. This process translates into a drop in myoelectric power from the muscle due to the loss in muscle conduction velocity. The k-means clustering technique (supervised k-means classifier algorithm) was used by Al-Mulla et al. [16] to visualize these three fatigue levels, dividing a heterogeneous population into a set of homogeneous groups of classes.

The EMG signal can be acquired both invasively (by using needle electrodes) and non-invasively (by placing electrodes on the skin surface). In order to avoid health and comfort problems to the users, surface electrodes are preferred. There are various non-invasive techniques in order to detect fatigue, such as ultrasound, electromyography, mechanomyography and near-infrared spectroscopy for both isometric and non-isometric contractions [1]. Surface electromyography (sEMG) is widely used for the evaluation of muscle activity and provides valuable information about localized muscle fatigue by means of changes in signal parameters [17,18,19], such as time domain parameters, frequency domain parameters, discrete wavelet transform and non-linear methods, inter alia. The sEMG signal is a one-dimensional non-stationary time series recorded from the muscle through an electrode collection [20]. The electrical activity of muscle fibres during contractions generates the sEMG signals, and the electrodes attached to the skin record these signals in a non-invasive manner [21]. Low-cost sEMG sensors have been widely applied in the rehabilitation field to control human motions [22,23,24]. In this work, surface electrodes are used to assess muscle fatigue, and the sEMG signal is acquired by a low-cost system.

It is important to highlight that most researchers on sEMG are focused on isometric contractions [1]. A muscle contraction is defined as isometric when the joint angle is constrained to be fixed and there are no changes in muscle length. In that case, the assessment of mean frequency or median frequency is the gold standard for muscle fatigue evaluation [25]. This is because the EMG frequency parameters are strongly associated with muscle fibre conduction velocity [9,26,27], and they are specifically sensitive to changes in the accumulation of metabolites (e.g., H^+^ and K^+^) at the local intramuscular environment [28]. The experiment performed in this work consists of such an isometric contraction of the quadriceps muscle group, specifically the rectus femoris muscle.

The EMG signal is described by its amplitude and frequency, because changes in these parameters happen when fatigue occurs [17,18,29]. During fatigue contraction, the sEMG amplitude is found to increase [30,31], but that change may not be significant. At the same time, there is a decrease in frequency parameters of the sEMG [32,33,34]. The sEMG signals acquired in this work are analysed both in terms of amplitude and time domain. Muscle characteristics differ from person to person. Therefore, there is no simple function of muscle load and timing that defines a precise muscle fatigue threshold [35]. The aforesaid notwithstanding, electric current measurement in a muscle can detect myoelectric manifestations of fatigue via changes in signal amplitude and frequency [36].

Despite sEMG being a common method in muscle fatigue research, this technique has some shortcomings, such as signal noise or incorrect electrode placement, inter alia. But there are ways to ameliorate these issues. There are algorithms that can reduce noise while maximizing the sEMG signal. Concerning the electrode placement, important for the reliability of signal, electrodes must be positioned in such a way as to minimize crosstalk with the signal from nearby muscles [37]. Research has led to proposed standards for placement to ensure repeatability. The European SENIAM (Surface ElectroMyoGraphy for the Non-Invasive Assessment of Muscles) project [38] proposed a standard electrode placement for 27 different muscles as well as recommendations for recording and processing sEMG signals. According to this proposal, an optimal electrode location exists for some muscles in the lower limbs in order to ensure that electrodes are positioned between the innervation zone and the tendon. This work has followed this protocol. In addition, several techniques validated by past research have been used to reduce signal noise, which will be explained later.

On another front, lack of exercise increases the risk of cardiovascular and chronic diseases as well as metabolic disorders. According to the World Health Organization [39], insufficient physical activity is one of the leading risk factors for death worldwide. Globally, one in four adults is not active enough. More than 80% of the world’s adolescent population is insufficiently physically active. Practising sports increases the health of blood vessels, and improves body oxygenation and muscle performance. Since fatigue is part of our daily life and considering the social and economic costs of injuries induced by overstraining of muscle, we decided to focus on studying muscle fatigue. As a first hypothesis, we want to analyze if people who are physically active are more fatigue resistant. This hypothesis seeks to validate the results, as it is known that physically active people are more fatigue resistant.

For this reason, making muscle fatigue detection easier and more accessible could lead to significant socio-economic benefits. At this point, the second hypothesis in this work arises. The question is whether it is feasible to use a low-cost system to detect muscle fatigue. Lastly, as a consequence of this previous point, the third hypothesis consists of proving if this low-cost implementation and equipment could obtain reliable and valid parameters and results of muscle fatigue detection.

## 2. Material and Methods

A testbed, in order to detect muscle fatigue, usually involves a volunteer subject performing a certain task, while the electromyographic signal is recorded by means of sensors placed on the skin. Afterwards, the acquired signal is post-processed and analysed. In this work, 28 healthy volunteer subjects performed a fatigue experiment where an isometric contraction of the quadriceps muscle group was undertaken with the help of a weight stack machine.

This section introduces the experimental approach adopted to test the hypotheses. Section 2.1 introduces the different elements that are part of the engineered testbed, from the questionnaire to the muscle low-cost signal acquisition equipment. Section 2.2 describes the experiment performed and explains the steps that have been followed. Finally, Section 2.3 deals with the techniques employed to generate results, providing an overview of signal filtering and the factors that characterise the evolution of fatigue along with isometric contraction.

### 2.1. Testbed Design

The testbed used for this experiment was split into three parts:

1. The first part was focused on getting some information about the volunteers related to their exercise habits, weight, body measurements, among others, by means of an online questionnaire.
2. The second part was the preparation of each volunteer for the experiment, where some pads were adhered to the skin of the leg using a specific palpation test.
3. Finally, the signal of the selected muscle was acquired.

#### 2.1.1. Questionnaire

In order to properly interpret the results of the sEMG signal, it is essential to be aware of the main characteristics of volunteers who participated in the experiment (age, gender, weight, height, physical activity, injury status and history, as well as smoke habits). To that aim, a questionnaire was prepared to be filled in online. Two main factors were highlighted, first the exercise habits of the subject and second the injuries he or she could have in his or her lower limbs.

It should be added that a criterion must be set for volunteer selection in order to obtain acceptable results. It is essential that the subjects be healthy because certain health factors, such as any diseases or pre-existing conditions, will greatly influence the fatigue outcome. Several studies exist which have looked at this influence. For example, smokers experience greater peripheral muscle fatigue [40]. On the other hand, sex and age factors also have an influence on muscle fatigue. Generally speaking, men are stronger than women, but less fatigue resistant during isometric contractions at submaximal and maximal intensities. Males may experience greater muscle fatigue than females at 40–60% of maximum voluntary contractions [41,42]. Older subjects show higher electromyographic activity levels [43].

A summary of all the information collected in this questionnaire is shown in the following table (Table 1).

#### 2.1.2. Preparation of Each Volunteer

As has been mentioned, 28 healthy volunteer subjects performed a fatigue experiment based on the isometric contraction of the quadriceps muscle group by means of a weight stack machine. The experiment protocol was explained to them and they signed an informed consent before their participation in this research study.

Electrode positioning plays a critical role in achieving accurate and repeatable estimates of sEMG signal amplitude, spectral variables and muscle fibre conduction velocity [44,45]. Several studies were consulted in order to identify the optimal electrode placement for muscles in the lower limbs. The optimal locations to place electrodes are the areas between the innervation zone and tendon terminations, where the EMG variables estimates are less affected by signal generation and by small electrode displacements [46].

Before starting the exercises to acquire the sEMG signal of the muscle, each volunteer was prepared fitted with electrodes on the Rectus Femoris muscle. Electrode placement was chosen by a palpation test of the area according to information available from an anatomical atlas [47].

Concerning the electrode placement, some researchers suggest standards for the placement to ensure repeatability. The European SENIAM project recommendations for sensor locations in upper leg muscles were applied in this work [38]. According to this proposal, the location of each electrode was chosen according to the following steps:

1. First, subjects were placed on a firm and plane surface.
2. By means of a palpation test, the iliac spine and the superior border of the patella were found.
3. A line between both points was drawn and the location of the electrodes selected. One close to the superior border of the patella, another on the line close to the belly and the last one on the side of the patella.

Proper skin preparation is necessary to reduce the electrode-skin impedance and to obtain a better fixation of the electrodes. Consequently, the area around the selected position was previously shaved with a disposable razor blade and cleaned with gauze and alcohol. Before placing the sEMG sensors the skin must be dry to reduce the skin impedance as low as possible [38].

SENIAM recommends applying the bipolar sEMG electrodes around the sensor location with an inter-electrode distance around 20 mm. In all experiments, this distance recommendation was considered. Also, the configuration was bipolar with the positive electrode close to the middle of the muscle and the negative close to the distal beginning of the rectus femoris. The reference electrode was placed on the knee joint.

#### 2.1.3. Muscle Sensor Signal Measurement

Different components to build a low-cost device where chosen with the ultimate goal of accurately measuring muscle response when it is involved in physical exercise. The main conditions to select those components were the price and their portability. The first condition was of utmost importance because one of the central objectives of the present study is to develop a measuring system able to obtain a reliable sEMG signal without spending too much money. The second condition was also vital because it is important to keep in mind the possibility of using this equipment in as wide a range of cases as possible.

Price comparisons with some commercial solutions are shown in Table 2, with some specifications described.

Following the aim of monitoring the behaviour of the muscle, a low-cost EMG sensor (Myoware muscle sensor) with the characteristics outlined in the following table (Table 3) was used.

This kind of sensors is designed to be connected with an Arduino board as an acquisition and processor system. There are different types of Arduino boards available in the market, but for the requirements of the present study, an Arduino board with the most powerful processing and memory characteristics as possible (Arduino MEGA) is necessary. The power supply of the sensor was built into the Arduino board within the necessary level of isolation. This configuration is safe for humans. Moreover, according to the instructions of the sensor manufacturer, this configuration is usually safe for the user and only in rare situations would a current loop to the electrical grid be created. In any case, previous test to check the safety of the system were carried out before starting the experiments with the volunteers.

Information related to the Arduino MEGA board can be found in Table 4.

In addition, a portable computer with Matlab/Simulink [48] was necessary to plug in the Arduino board and store the acquired data. To that aim, Simulink had to be configured in a specific way. First of all, and taking into account that this experiment is a first approximation of a future portable EMG system, the data was stored in the computer instead of in the Arduino board. Simulink stop time simulation was configured as infinite and communication as an external connection. Also, the hardware board was defined as Arduino Mega 2560 to deploy the code. This data will be useful for future analysis.

**Figure 1:**
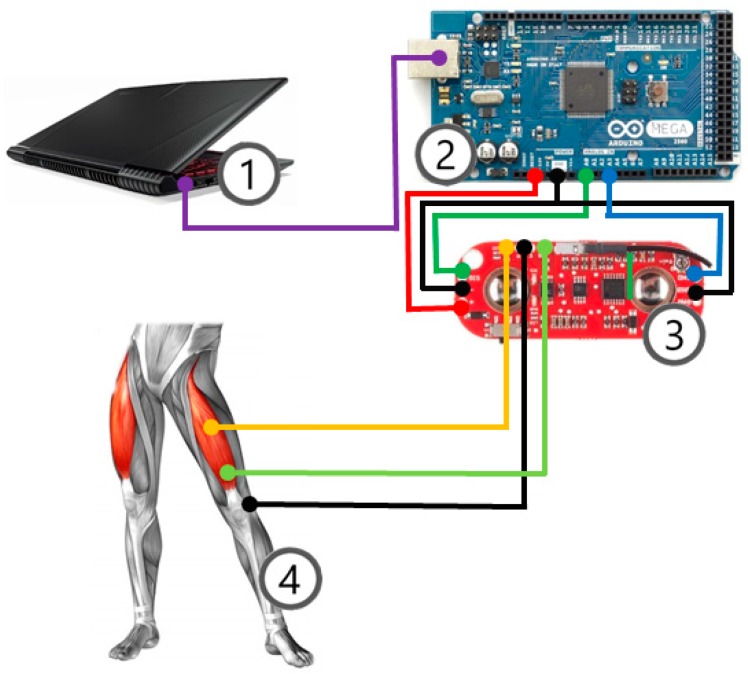
Setup wire configuration: **①** personal computer, **②** Arduino MEGA board, **③** surface electromyography (sEMG) sensor by Myoware System, **④** volunteer.

A diagram of the connections between components is shown in Figure 1.

Finally, according to the anatomical references [49], electrodes with the characteristics shown in Table 5 were placed on the leg following the instructions of different studies [38].

### 2.2. Experiment

**Figure 2:**
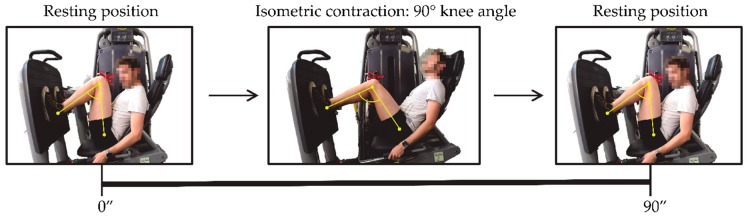
Control position of the volunteers while performing the isometric contraction of the rectus femoris.

The experiment was based on the acquisition of the EMG signal while the volunteers performed an isometric contraction of the quadriceps muscle group using a weight-stack machine to strengthen the lower limbs. The applied load in the exercise was 30 kg, and it was sustained by all the volunteers. The position adopted by the subjects during the exercise is shown in the picture below (see Figure 2). From a rest position (at zero seconds), with feet placed in parallel and aligned with the hip, and the back against the backrest, the subject adopts a posture in which the knee angle (between femur and tibia) is approximately 90 degrees. The volunteer should maintain this 90-degrees position (rectus femoris isometric contraction) for 90 s. After that, the subject recovers his or her rest position. Each volunteer was guided along the exercise in order to ensure they adopt correct and not injurious positions. All subjects were coached to adopt an appropriate posture in order to activate the rectus femoris until they felt muscle fatigue.

Also, with the main idea of being able to follow the evolution of fatigue in the quadriceps muscle group, other studies were previously checked [50,51] to set the minimum time for the appearance of muscle fatigue. Therefore, it was considered that the isometric contraction should last 90 s.

**Figure 3:**
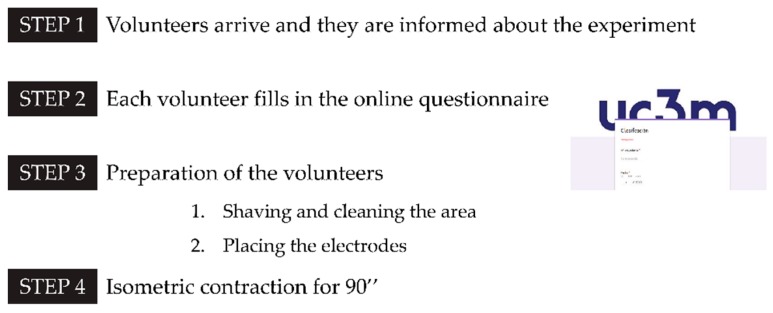
Steps of the experiment and a short explanation of them.

The following diagram (Figure 3) represents the different steps that each volunteer completed when he or she performed the experiment.

Step 1. Once the volunteer arrived at the facilities where the experiment was carried out, he or she was informed about the risks and how the experiment would unfold. After each volunteer had understood everything and agreed on the rules, he or she passed to the next step.

Step 2: According to Section 2.1.1, some background information was necessary to put the experiment into practise. For this reason, subjects were asked to fill in an online questionnaire about their habits and physical features.

Step 3: In order to successfully measure the electromyogram signal from the muscles, the hair of the area where the electrode would be placed must be removed and the skin must be clean and dry. The hair was shaved with a disposable razor. Afterwards, a sterile gauze with alcohol was used to wipe the area. Once the area was completely dry, the electrodes were adhered onto the skin. Also, the main joints of the lower limb (hip, knee and ankle) were highlighted with yellow stickers to control and measure the angle of the knee. An example of the knee angle measurement is shown in Figure 2. In order to avoid injurious positions for the subjects, and with the aim of performing correctly the isometric contraction of the rectus femoris, the knee angle adopted in this machine must be approximately 90 degrees.

Step 4: The last step was the actual performance of the proposed exercise, the isometric contraction. The main purpose of this step was to pinpoint when each volunteer felt the quadriceps muscle fatiguing. All this could be done thanks to the testbed described in Section 2.1 which is made up of a personal computer, low-cost acquisition equipment and low-cost sEMG sensors. All of this was connected and programmed via Matlab and Simulink [48].

### 2.3. Signal Processing Methods for Muscle Fatigue Evaluation

Once the exercises were finished and the sEMG signal stored in the computer, it was time to process the signal to get the fatigue evolution of each volunteer. For that aim, it was necessary to follow a series of steps. First, the sEMG signal must be filtered in order to reduce the noise and, after that, a few indicators of the fatigue evolution were estimated.

On the one hand, the use of wavelet analysis has become more common [52] in the denoising of the sEMG signal. Wavelet analysis is not affected by the non-stationary properties of sEMG signals [53].

On the other hand, as previously mentioned, the identification of muscle activity can be assessed based on sEMG signals by examining the amplitude and frequency. Amplitude is typically evaluated by the root mean square of the sEMG signal or the integrated area under the curve. Moreover, in sEMG signals recorded during isometric contractions, there are changes in the properties of the spectral sEMG signal that occur for several seconds. Various features will be extracted to obtain meaningful information from the sEMG data.

#### 2.3.1. Signal Filtering

Due to the kind of signal, there are various noises that should be eliminated before analysing the signal. All those noises can come from the adjacent muscles, from the background, from the chemical composition of the skin and also from movement, inter alia.

Researchers have made strenuous efforts to solve the problem of EMG signal denoising [54,55,56,57,58]. Various digital signal processing techniques are employed, from classical digital filters to modern filtering techniques such as wavelets. The wavelet transform decomposes a signal into numerous multi-resolution components and it is used to detect and characterize the short time component within a non-stationary signal and provides information regarding the time-frequency of the signal [59,60]. Wavelet transforms can be used to characterize the localized frequency content of each motor unit action potential [61,62]. Therefore, wavelet transform allows extracting features from the EMG signals. The most used analysis method is the discrete wavelet transform (DWT) [2].

In sEMG signal analysis, the use of wavelets has become more usual [53] because it applies certain tools that change the time resolution for each frequency. In this way, it gives a higher time result for high bands and a lower resolution time for low-frequency bands. This method provides better time and frequency approximation to the physiological signals if compared to the Fourier short transform procedure, where the resolution for each band is always the same.

Wavelet denoising has the advantage over traditional filtering of removing noise at all frequencies and can be used to isolate single events that have a broad power spectrum or multiple events that have varying frequency. Therefore, wavelets can be used to reduce the signal to noise ratio whilst retaining the fatigue content. However, a methodical process to select the proper wavelet does not exist. The wavelet selection depends on the signal type (sEMG signal in this case) and the intended purpose of this technique (the aim with this technique in this work being denoising). According to that, the wavelet family which most closely matches the sEMG signal will be chosen.

The wavelet technique allows denoising without smoothing out the sharp structures, which results in a cleaned-up signal that still shows important details. For time series where an aperiodic shift in the time series is expected, as in this case, we need an orthogonal wavelet. The use of an orthogonal basis implies the use of the discrete wavelet transform. Several studies [52,53,58,60], in which the analysed signal was similar to this study, were consulted in order to establish a first approximation of which wavelet functions could fit better in this work. On the basis of that, different wavelet functions (in particular, Daubechies and Symlets wavelets) with different number of decomposition levels were checked in this study. This comparison showed that the best wavelet functions for this application were symlets wavelets, specifically sym8 wavelet function with a 12-level wavelet decomposition and the thresholding rule for the denoising method was Bayes-Mean. For this comparison, we used the Wavelet Toolbox of Matlab (Wavelet Signal Denoiser), which allows you to visualize and automatically denoise time-series data. By means of this app, one can modify the wavelet function, the number of decomposition levels, the thresholding strategies, among other denoising parameters, and finally compare results inspecting the denoised signal, their coefficients and the signal-to-noise ratio.

#### 2.3.2. Estimation of the Fatigue Indicators

The reliability of the pattern classification system depends on the choice of features used to represent the raw EMG signals [63]. Many authors combine various sets of sEMG parameters based on the idea that using traditional variables alone is not sensitive enough to determine muscle fatigue. It is desirable to select several feature parameters for EMG pattern classification since it is very difficult to extract a single feature parameter that shows the distinctive feature of measured signals [64].

According to some studies [35,65], the assessment of some indicators based on the time domain method can be used in order to represent muscular effort and fatigue depending on the change of the sEMG signal.

In the present study, three indicators were calculated, two related to the amplitude and a third one related to the mean frequency.

By drawing on the concept of the effect of amplitude, Knowlont et al. [66] confirmed that an increase in signal amplitude implies the appearance of muscular fatigue. For that reason, two indicators of the sEMG amplitude were assessed: MAV (mean absolute value) Equation (1) and RMS (root mean square) Equation (2) value.

(1) $$MAV=\frac{1}{N}\overset{N}{\underset{i=1}{\sum }}\left|x_{i}\right|$$

(2) $$RMS=\sqrt{\frac{1}{N}\overset{N}{\underset{i=1}{\sum }}x^{2}}$$

*N* is the number of samples and *x_i_* the value of the signal in the *i* sample.

Moreover, Mario Cifrek et al. [19] indicates that EMG signal amplitude is not usually used as an isolated indicator of muscle fatigue. For that reason, a third indicator (mean frequency (MNF)) was assessed Equation (3).

(3) $$MNF=\frac{\sum_{j=1}^{M}f_{j}P_{j}}{\sum_{j=1}^{M}P_{j}}$$

*f* is the frequency value of the EMG power spectrum in frequency interval *j*, *P_j_* is the EMG power spectrum in frequency interval *j*, and *M* is the length of the frequency interval.

In contrast with the amplitude indicators, a decrease in the result of the MNF implies the appearance of muscle fatigue.

All those signal treatments have been programmed by means of a script in Matlab with a time window of 1 s and no overlap. That script was run after each test and the results are presented in the following section.

## 3. Results

The information gathered in the experiment is presented below. First, we will look at the information related to the volunteers’ classification, and later the EMG signal and the indicators needed to define the behaviour of muscle fatigue.

### 3.1. Volunteer Classification

The results obtained from the preliminary questionnaire are summarized in this section. Twenty eight volunteers participated in the study, of whom 54% were women and 46% men. The main classification parameters, such as age, height and weight, are shown in Table 1.

In order to ascertain if the sample used in the analysis was appropriate, a descriptive statistical analysis was carried out in which a 95% confidence interval was set. Results showed that the sample meets the null hypothesis of normal distribution, making it possible to confirm the case of normality in the procedure

### 3.2. Surface Electromyography (sEMG) Signal and Evolution of Their Indicators.

In this section we present the EMG data collected during the experiments and the results of the indicators used to identify the behaviour of fatigue on each of the participants.

**Figure 4:**
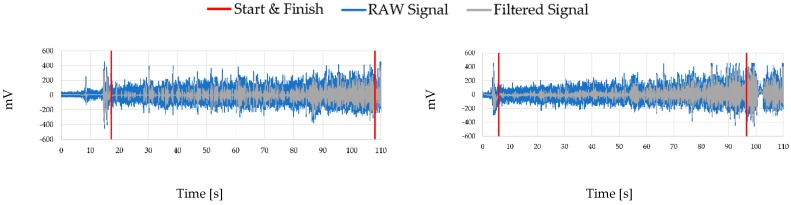
Example of the raw electromyography (EMG) signal amplitude (mV) captured in the experiment across time (s), where it can be observed how the amplitude increases in time. The example on the bottom comes from a female who is 24 years old, exercises 3 h a week and has a BMI of 24, and the example on top comes from a male who is 23 years old, exercises 3 h a week and has a BMI of 23.6.

Figure 4 shows the raw and filtered EMG signal of two volunteers while they performed the isometric contraction. What stands out is how the amplitude of the signal increases in time, a factor that is supported by other indicators. This can mean that the muscle is actively working and feeling fatigued. Moreover, as it was explained in a previous section, each signal must be filtered before we can assess its indicators. For that reason, filtering by wavelets was applied to each experiment. 

**Figure 5:**
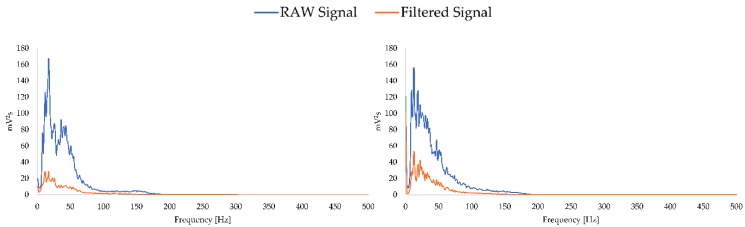
Signal spectrum of the EMG signal before and after filtering. The example on the left comes from a female who is 24 years old, exercises 3 h a week and has a BMI of 24, and the example on the right comes from a male who is 23 years old, exercises 3 h a week and has a BMI of 23.6.

In addition, in Figure 5, for a clearer picture of the effects after the signal has been filtered, the frequency spectrum before and after filtering is shown below for the same examples as in the previous figure.

**Figure 6:**
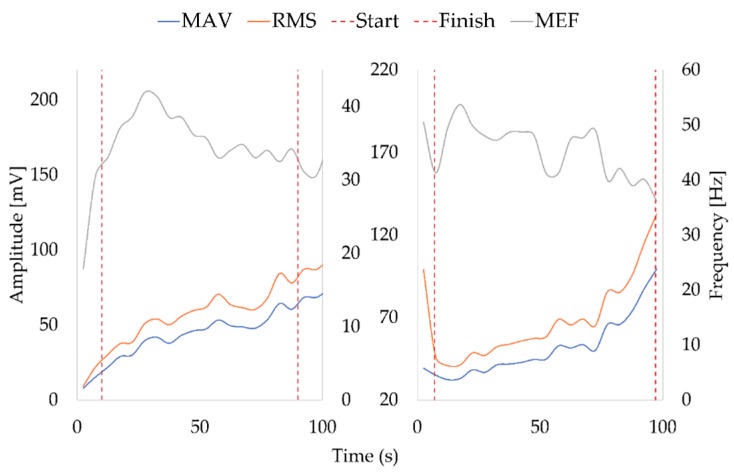
Two time cropped examples of the root mean square (RMS, left vertical axis), mean absolute value (MAV, left vertical axis) and mean frequency (MNF, right vertical axis) across time (s). The example on the left comes from a female who is 24 years old, exercises 3 h a week and has a BMI of 24, and the example on the right comes from a male who is 23 years old, exercises 3 h a week and has a BMI of 23.6.

Once the signal was filtered, the MAV, RMS and MNF indicators were assessed by means of a programmed Matlab script. These lines show results from two volunteers, a male and a female (Figure 6).

On one hand, we can see how in both examples the average frequency decreases over time, with a relatively stable slope. On the other hand, we can observe how the slopes of the MAV and RMS increase. All these parameters show how the muscle in both examples is experiencing fatigue, but looking at each one separately it is not possible to determine the moment precisely. For that reason, in the present work, time fatigue assessment has been accomplished by means of the three indicators (MAV, RMS and MEF). Moving ahead with the examples in Figure 6, it can be seen how the slopes of the MAV and the RMS change at around 70 and 75 s for both examples. By contrast, the MEF shows how the slope becomes slightly negative from this point for the sample on the left and strongly negative for the sample on the right. Later it is observed that for both examples the slopes level out again. This behaviour happens because the muscles around the quadriceps attempt to support it because it, itself, starts to suffer fatigue.

### 3.3. Fatigue Evolution

**Figure 7:**
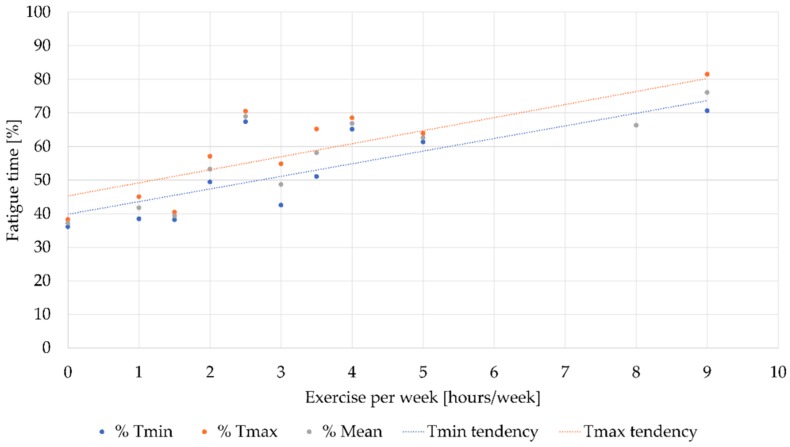
Tendency of the fatigue time versus exercise per week. The results of the experiments were grouped on the basis of the number of hours of weekly exercise in order to evaluate the maximum, minimum and average value. These values are represented in this figure.

Now we move on to the results obtained after carrying out the exercises of the experiment and using the analysis methods outlined above. The results were plotted in Figure 7. This figure represents the percentual time in which muscle fatigue appeared in each of the volunteers, represented by the letter T and a sub-index for each of the computed variables (min: minimum and max: maximum). This time was the fraction between the moment in which fatigue was detected and the duration of the isometric contraction. In addition, volunteers were grouped based on the number of hours of exercise they practised weekly.

Qualified personnel in muscle fatigue analysis were consulted in order to compute the fatigue time. Moreover, the instant of time in which the subjects manifested to begin to feel the sensation of muscle fatigue was registered. This instant of time was later contrasted with the moment of time in which the parameters analysed experienced changes. It should be mentioned that the test is anonymous, and the volunteers cannot be identified.

The tendency to fatigue has been assessed by linear regression, the best option to calculate tendency in this case in order to obtain clear results.

Data analysis was used to group together volunteers who spend the same number of hours per week exercising, where there is an especially high number of subjects who practise less than two hours of exercise per week (Table 6).

As can be seen, most volunteers exercise between 0 to 3 h a week. Furthermore, there is a clear relationship between the number of hours of exercise and the muscular capacity against fatigue. It is important to highlight how the tendency for both variables is to increase as any of them rise.

In addition, the following table (Table 7) includes slightly more precise information on each of the values taken into account.

It is important to underline that results shown in this table have been represented on a percentage basis due to the fact that some volunteers exceeded for a few seconds the duration of the exercise, even though the duration was set to 90 s and warnings at the beginning and at the end were included.

## 4. Discussions

There are many references [1,28,67,68,69,70,71,72,73] in which sEMG signals were used to characterize muscle fatigue. Most studies reported no significant difference between electromyographic and metabolic thresholds. There is a great variety in study protocols, measurement techniques and data processing. Furthermore, threshold detection from the quadriceps femoris muscle group (vastus medialis, vastus lateralis, and rectus femoris) is recommended [67]. Evaluating the status of the muscle while a person performs an exercise or a task can prevent muscular overstraining and, consequently, possible injuries. In today’s market there are several commercial and advanced solutions that provide the possibility to precisely measure EMG signals of the muscles, but at a high cost. This article describes a low-cost system developed for the detection of muscle fatigue in order to make this technique more accessible.

All this was carried out using low-budget hardware, comprising an Arduino MEGA board and an EMG Myoware sensor. The whole system was programmed and communicated with a personal computer that loaded a code especially designed using Matlab and Simulink. Figure 1 shows a diagram of the system.

Once a fully-functional device was set up, a number of checks were performed to see if the device met the desired requirements and it was able to properly detect muscle fatigue. The test consisted of a 90-s isometric contraction of the quadriceps muscle group using a weight-stack machine with an applied load of 30 kg. The reaction of the rectus femoris muscle was recorded during that time by means of the designed device, later analysed through the data collected and checked to see if the designed low-cost system meets the requirements.

A group of 28 healthy volunteers, aged between 20 and 35 years, of whom 48% were men and 52% women, were recruited for this trial. All of them were kindly invited to complete an online questionnaire where they were asked about their exercise habits (weekly hours of exercise, sports practiced …), injury status and history, smoking habits, and main physical characteristics, resulting in a distribution as shown in Table 1.

As can be seen, although men have a slightly higher BMI score than women, all volunteers are within the healthy range (18.5–24.9). It is also evident that the average number of hours that both groups exercise is above two hours per week, with only a single weekly exercise event. It is also important to highlight that the value of the standard deviation is almost equal to the average value. This is because a high number of participants have a completely sedentary life (Figure 6).

All these data suggest that volunteers who exercise less per week will be those in whom fatigue will appear earlier. The first hypothesis (people who are physically fit are more fatigue resistant) was really used in this study in order to verify the results obtained by the low-cost EMG system. It has already been proven that people who exercise regularly are more fatigue resistant. With this in mind, our results should show the same conclusion.

Once we knew the characteristics of the experimental population, and after checking that the distribution of the results fulfilled the null hypothesis, the isometric contraction exercise was performed by the subjects in order to acquire the EMG signal generated by the rectus femoris from the muscular group of quadriceps.

Due to the fact that the sEMG signal carries a significant noise output, the signal was filtered by means of wavelets.

Once all the results were available, a series of estimators were calculated in order to be able to trace the muscular activity and to estimate the moment in which fatigue appeared. These estimators have been calculated by means of a mathematical Matlab program. MAV and RMS reflect the appearance of fatigue when their values rise. On the other hand, the mean frequency indicator behaves in the reverse. When a slope change is noticed, fatigue appears. An example of the evolution of those parameters can be seen in Figure 6. Furthermore, results were confirmed with the expressed sensations of the volunteers during the experiment. The instant of time in which each volunteer manifested a sense of fatigue was recorded and compared to the results in order to confirm the indicator changes in the graphs at specific seconds.

Most researchers analyse sEMG signals in terms of time-amplitude and frequency domains. The time domain features are the most popular because of their computational simplicity. For raw EMG signal analysis, the average rectified value (which measures the average of the absolute signal value) and the root mean square (which measures the signal power) are typically used [1,74]. De Luca [28,75], who is a reference guide in the field of EMG, defines these last two parameters as appropriate analysis methods. The power spectrum density also reveals muscle fatigue when the frequency content of the signal is compressed proportionally [76]. Frequency-domain features are mostly used to study muscle fatigue and to recognize movements.

The amplitude of sEMG signals is influenced by the number of active motor units [77], their discharge rates, and the shape and propagation velocity of the intracellular action potentials [78]. Research on sEMG concludes that an amplitude increase in EMG signal or shifts in the spectrogram suggest signs of muscle fatigue in static contractions [28,79,80,81,82]. Other works about muscle fatigue during isometric contractions under controlled conditions have established typical sEMG readings in spectral parameters [1]. These spectral parameters are related to changes in muscle fibre conduction velocities and subsequent changes in the duration of the motor unit action potential waveform [83]. Petrosky et al. [18] show a decrease in the centre frequency for all muscle groups. It has been shown that during static contractions, the mean frequency usually decreases [77,84,85,86]. Other authors [17] also show changes in amplitude and median frequency when muscles fatigue. The results of this work show the same behaviour of the sEMG signal when fatigue appears as previous studies. Therefore, this confirms our third hypothesis. In other words, it is possible to obtain reliable results using a low-cost system in order to detect muscle fatigue.

The same procedure was applied to all tests. Considering the results shown in Table 6, it can be seen that the first hypothesis is fulfilled, because the volunteers who were more physically active needed more time to feel fatigue, and it did not matter whether they were men or women. In addition, taking into account that fatigue occurs mostly when more than 40% of the test time has elapsed, with the earliest case being 26% and the latest being 83%, a summary table is presented below to better describe this distribution.

This table (Table 8) shows, in percentage terms, the number of people who were in a certain level of physical condition, under 50 s signifying a low physical level, between 50 and 60 a medium physical level, and over 60 a very good physical level.

In addition, we can see how the allocation of volunteers between both tables behaves in a very similar way, reaffirming the first hypothesis and thus proving the effectiveness and behaviour expected of the system deployed.

Despite shortcomings of sEMG, the low-cost sEMG system and procedure presented in this study for muscle fatigue assessment may hold some clinical utility, although it is important to understand the limitations of the chosen tool.

It is essential to know how to place the EMG sensors to get a low noise signal. It is also important to note that when a fully onboard system is available, the signal will be less noisy.

The current research study recorded and analysed the sEMG data performed only by healthy subjects. It is essential that the subjects be healthy because certain health factors (such as any diseases or pre-existing conditions) will greatly influence the fatigue outcome.

The use of sEMG requires knowledge of how the signal is generated. Even though the acquisition of a signal is moderately easy, inaccurate findings are easily obtained when inappropriate methods are used.

## 5. Conclusions

According to the results, the following conclusions can be asserted within this study.

The method used for fatigue detection is valid because it provides results that are reasonable and easily recognisable. In addition, the entire program has a very small computational weight, which allows it to be installed in a low-cost system such as the one used in this study, which supports the second hypothesis expressed at the beginning of the article.

The third hypothesis is considered valid with the results being in accordance with hypothesis one. The system is valid as the results meet the expectations in hypothesis one, not only because a low-cost hardware system has been used, but also because implementation using Simulink and the acquisition system (Arduino) reported good results.

## Figures and Tables

**Table 1:** Main characteristics of the subjects who participated in the study.

Genre	Age	Active Exercise (hour/week)	BMI *	Height (cm)	Weight (kg)
Female	23.7 ± 4.5	2.2 ± 2.4	21.4 ± 2.8	166.1 ± 8.5	59 ± 11.4
Male	24.5 ± 9.3	2.4 ± 3.1	23.4 ± 2.7	174.5 ± 6.7	71.4 ± 10.5

* Body mass index.

**Table 2:** Comparison between different commercial systems and the custom low-cost system and its technical data (Approved by: product is safe according to the applicable directives (CE “*Conformité Européenne*”). Classified as: product was designed to be used as … Isolated: if the product has internal electrical isolation, answer is “Yes”; but if the product needs external isolation, answer is “No”).

**Plug and Play Devices**				
Equipment	Price (€)	Approved by	Classified as	Isolated
Delsys Trigno	(around) 20,000	CE	Medical device	Yes
Cometa	(around) 15,000	CE	Biomedical device	Yes
biosignalsplux Explorer	Up to 1000	CE	Research	Yes
NeuroNode	Up to 14,000	CE	Wearable	Yes
**Customize devices**				
Equipment	Price (€)	Approved by	Classified as	Isolated
Bitalino	Up to 150	CE	Research device	Not
Myoware EMG + Arduino MEGA *	100	CE	Research device	Not

* Device used in the present paper.

**Table 3:** Technical data of the Myoware Muscle Sensor (AT-04-001). CMRR (Common Mode Rejection Ratio).

Technical data Myoware Muscle Sensor	
Size	0.82″ × 2.06″
Output modes	EMG Envelope/Raw EMG
Input impedance (GΩ)	110
Gain	201R_gain_/1 kOhm
Bandwidth (Hz)	10–400
CMRR (dB)	110

**Table 4:** Technical data of the Arduino MEGA. SRAM: Static RAM, ADC: Analog Digital Converter.

Microcontroller	ATmega2560
V_in_ (V)	7–12
V_out_ (V)	6–20
Diginal Inputs/Outputs	54
Analogue Inputs	16
Flash Memory (Kb)	256
SRAM (Kb)	8
ADC (bits)	10
Clock Speed (MHz)	16

**Table 5:** Technical data of the EMG electrodes.

Technical data EMG electrodes	
Shape/Size (Excl. Grip)	Round/⦰ 24 mm
Gel area	201 mm^2^
Adhesive area	251 mm^2^
Sensor area	80 mm^2^
Product thickness (adapter excluded)	1 mm
Gel characteristics	Conductive and adhesive hydrogel
Sensor	Polymer Ag/AgCl coated

**Table 6:** Distribution (%) of the volunteers according to the time they spend practising sport along the week.

Exercise time	≤ 2 hours	> 2 & < 5 hours	≥ 5 hours
Male	32%	4%	11%
Female	32%	14%	7%

**Table 7:** Summary of the information collected from the experiments.

Genre	Age (years)	Active exercise (μ ± σ) (hour/week)	T_max_/T_min_ (%) *	Time (μ ± σ) (%) *
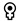	23.7 ± 4.5	2.2 ± 2.4	54.1/49.2	51.6 ± 14.8
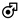	24.5 ± 9.3	2.4 ± 3.1	51.7/46.1	48.9 ± 13

* T_max_/T_min_: maximum and minimum time when fatigue appears according to the duration of the exercise (90″), Time: mean and standard deviation of the moment when fatigue appears.

**Table 8:** Distribution (%) of the volunteers according to the time they started feeling muscle fatigue.

Fatigue Time	≤ 50 s	> 50 & < 60 s	≥ 60 s
Male	29%	7%	11%
Female	25%	14%	14%
